# Anionic metabolite biosynthesis enhanced by potassium under dark, anaerobic conditions in cyanobacteria

**DOI:** 10.1038/srep32354

**Published:** 2016-08-31

**Authors:** Sakiko Ueda, Yuhki Kawamura, Hiroko Iijima, Mitsuharu Nakajima, Tomokazu Shirai, Mami Okamoto, Akihiko Kondo, Masami Yokota Hirai, Takashi Osanai

**Affiliations:** 1 School of Agriculture, Meiji University, 1-1-1, Higashimita, Tama-ku, Kawasaki, Kanagawa 214-8571, Japan; 2 RIKEN Center for Sustainable Resource Science, 1-7-22 Suehiro-cho, Tsurumi-ku, Yokohama, Kanagawa 230-0045, Japan; 3 Department of Chemical Science and Engineering, Graduate School of Engineering, Kobe University, 1-1, Rokkodai, Nada, Kobe 657-8501, Japan

## Abstract

Potassium (K^+^) is an essential macronutrient for all living organisms including cyanobacteria. Cyanobacteria are a group of bacteria performing oxygenic photosynthesis, widely studied in basic and applied sciences. The primary metabolism of the unicellular cyanobacterium *Synechocystis* sp. PCC 6803 is altered by environmental conditions, and it excretes organic acids and hydrogen under dark, anaerobic conditions. Here we demonstrated that K^+^ widely changes the primary carbon metabolism of this cyanobacterium. Succinate and lactate excretion from the cells incubated under dark, anaerobic conditions was enhanced in the presence of K^+^, while hydrogen production was repressed. The addition of K^+^ and the genetic manipulation of acetate kinase AckA and an RNA polymerase sigma factor SigE additively increased succinate and lactate production to 141.0 and 217.6 mg/L, which are 11 and 46 times, compared to the wild-type strain without K^+^, respectively. Intracellular levels of 2-oxoglutarate, succinate, fumarate, and malate increased by K^+^ under dark, anaerobic conditions. This study provides the evidence of the considerable effect of K^+^ on the biosynthesis of anionic metabolites in a unicellular cyanobacterium.

Cyanobacteria are a group of oxygenic photosynthetic bacteria important for carbon and nutrient cycles, and contributing at least 25% of global primary productivity^1^. Cyanobacteria are used as the chassis —a sort of “green *Escherichia coli*”— for synthetic biology because they are responsive to genetic modification^2^,^3^. Genomic information on cyanobacteria is available on public databases such as CyanoBase (http://genome.microbedb.jp/cyanobase/) and CYORF (http://cyano.genome.jp/).

Bacterial cells substantially alter their primary metabolism and produce various metabolites during fermentation^4^. Cyanobacteria including *Synechocystis* sp. PCC 6803 (hereafter *Synechocystis* 6803) produce hydrogen under dark, anaerobic conditions^4^,^5^,^6^. This hydrogen coordinates energy expenditure and primary metabolism^7^. Under dark, anaerobic conditions, *Synechocystis* 6803, *Synechococcus* sp. PCC 7002, and *Arthrospira maxima* cells are known to excrete organic acids and/or amino acids^8^,^9^,^10^. *Synechococcus* sp. PCC 7002 wild-type cells degrade intracellular glycogen, and produce lactate, alanine, and acetate under dark, anaerobic conditions^8^. *Synechocystis* 6803 produce organic acids such as acetate, succinate, and lactate^10^. Acetate is mainly synthesized by an acetate kinase, AckA^10^. Genetic manipulation by *ackA* knockout and the overexpression of *sigE*, which encodes an RNA polymerase sigma factor, SigE, increased lactate and succinate production during dark, anaerobic incubation^10^. Another study showed that co-overexpression of heterologous lactate dehydrogenase and pyruvate kinase in *Synechocystis* 6803 increased the L-lactate production under continuous light conditions^11^,^12^. Consequently, increased interest in the hydrogen and organic acid productions in cyanobacteria aims to increase potential to produce valuable products by direct conversion of CO_2_.

The potassium ion (K^+^) is the most important and abundant cation in almost all types of the cells. The cytosolic K^+^ concentration lies within a 100–200-mM range in plant cells, and increases up to ~1 M in bacterial cells^13^,^14^. K^+^ functions in electrical neutralization of anionic charges, pH homeostasis, protein synthesis, control of membrane polarization and regulation of osmotic pressures^15^,^16^. K^+^ is also indispensable for metabolic enzyme and molecular chaperon activation^17^. K^+^ is involved in phosphoryl transfer reactions during primary metabolism by binding to the phosphate backbone of nucleic acids^17^. The regulation of K^+^ transport across the thylakoid membranes importantly contributes to the maintenance of photosynthesis and respiratory electron transport in cyanobacteria; it is particularly important under photomixotrophic conditions^18^. K^+^ deficiency causes a pleiotropic effect on cyanobacteria: a decrease in photosynthetic pigments and activity, change in protein profiles, and aberrant cell volumes after hyperosmotic shock^19^,^20^. K^+^ is thus known to alter various aspects of the cyanobacterial cells at physiological, molecular, and biochemical levels, although a metabolomic analysis has not been performed.

We examined the effect of K^+^ on primary metabolism in *Synechocystis* 6803 under dark, anaerobic conditions and demonstrated that K^+^ enhanced the biosynthesis of anionic metabolite both intracellularly and extracellularly.

## Methods

### Bacterial strains and culture conditions

The glucose-tolerant (GT) strain of *Synechocystis* sp. PCC 6803, isolated by Williams^21^, was grown in modified BG-11 medium, consisting of BG-11_0_ liquid medium^22^ and supplemented with 5 mM NH_4_Cl (buffered with 20 mM HEPES–KOH, pH 7.8). Of the GT substrains, we used the GT-I strain was used in the current study^23^. The 1299E strain, lacking acetate kinase and overexpressing *sigE*, had been generated previously^10^. Liquid cultures were bubbled with air containing 1% (v/v) CO_2_ (the flow rate was 30~50 mL/min) and incubated at 30 °C under continuous white light (~50–70 μmol photons m^−2^ s^−1^). Modified BG-11 medium (containing 10 mM NH_4_Cl in liquid medium) was solidified with agar (1.5% w/v) for plate cultures, and incubated in air at 30 °C under continuous white light (~50–70 μmol photons m^−2^ s^−1^). Cell densities were measured at *A*_730_ using a Hitachi U-3310 spectrophotometer (Hitachi High-Tech., Tokyo, Japan).

### Dark, anaerobic incubation

Cells grown in 70 mL of modified BG-11 medium (started from *A*_730_ = 0.4) for 3 days were concentrated into 10 mL of HEPES buffer (20 mM HEPES–KOH, pH 7.8) to *A*_730_ = 20 in a GC vial (cells in 50~60 mL culture were harvested). The vial was sealed using butyl rubber, and N_2_ gas was introduced using syringes for 1 h to generate anaerobic conditions. After removing the syringes, the vial was wrapped with aluminum foil and shaken at 30 °C on a laboratory shaker at 180 rpm. Cell cultures were then centrifuged at 5,800 × *g* for 2 min, the supernatant was filtrated, and 1 mL of supernatant was freeze-dried per day. The dried sample was used for high-performance liquid chromatography (HPLC) analysis.

### Measurement of excreted organic acids by HPLC

Freeze-dried supernatants were dissolved in 100 μL of filtered 3 mM perchloric acid. The dissolved samples were analyzed by HPLC using a LC-2000Plus Systems (JASCO, Tokyo, Japan) with a photodiode array detector and two RSpak KC-811 columns (Showa Denko, Tokyo, Japan). Organic acids were quantified with 0.2 mM bromothymol blue in 15 mM sodium phosphate buffer; peaks were detected at 445 nm. The column temperature was 60 °C, and the flow rates of 3 mM perchloric acid and 0.2 mM bromothymol blue solutions were 1.0 mL/min and 1.5 mL/min, respectively. Powders of citrate, succinate, lactate, and sodium acetate were used as standards and purchased from Wako Pure Chemicals Co. Ltd (Osaka, Japan).

### Gas chromatography-thermal conductivity detector (GC-TCD) analysis

Cells were incubated under dark, anaerobic conditions as wells as the experiment of organic acid production. The accumulated H_2_ gas in the headspace of a GC-vial was measured with a gas chromatograph (GC-2010 Plus AT, Shimadzu, Kyoto, Japan) as described previously^24^ and in accordance with the manufacturer’s instructions. N_2_ was used as the carrier gas with a flow rate of 10 mL/min.

### Liquid chromatography mass spectrometry (LC-MS/MS) analysis

Cells were incubated under dark, anaerobic conditions as wells as the experiment of organic acid production. Equal amounts of cells (10 mL cell culture with *A*_730_ = 1.0) were harvested by rapid filtration before and after dark, anaerobic incubation for 3 days. Metabolites were extracted using a previously described method^25^. The harvested cells were filtered, and the metabolites were rapidly extracted in 1.2 mL solvent mixture (CHCl_3_:CH_3_OH:H_2_O, 2.5:2.5:1, v/v/v) with 10 μg/L D-(+)-camphor-10-sulfonic acid as an internal standard. After centrifugation at 15,000 × *g* at 4 °C for 5 min, 400 μL of the upper phase was transferred to a new tube and vacuum-dried. The metabolites were quantified by high-performance liquid chromatography coupled with electrospray ionization tandem mass spectrometry (LCMS-8040 triple quadrupole LC/MS/MS spectrometer; Shimadzu, Kyoto, Japan).

### Gas chromatography mass spectrometry (GC-MS) analysis

Cells were incubated under dark, anaerobic conditions as wells as the experiment of organic acid production. Equal amounts of cells (10 mL cell culture with *A*_730_ = 1.0) were harvested by rapid filtration as with LC-MS/MS analysis. GC-MS was carried out using a GCMS-QP2010 Ultra (Shimazdu), the detailed protocol of which is described by Osanai *et al*.^26^. A CP-Sil 8 CB-MS capillary column (30 m × 0.25 mm × 0.25 μm; Agilent, Palo Alto, CA, USA) and helium were used for metabolite separation and as the carrier gas, respectively (flow rate is 2.1 mL/min). Each 1 μL sample was injected with a spilt rate of 1:10. An oven temperature was maintained for 10 min at 60 °C, then raised to 315 °C at 15 °C/min, and maintained for 6 min. The other equipment was set at 250 °C interface temperature, 200 °C ion source temperature, and electron impact ionization (EI) at 70 eV. Organic acids were derivatized for 90 min at 30 °C in 20 μL 20 mg/mL methoxyamine hydrochloride in pyridine, and trimethylsilylationwas performed for 30 min at 37 °C, and for 2 h at room temperature with 50 μL N-methyl-N-(trimethylsilyl)trifluoroacetamide. Pimelate was used as an internal standard.

### Statistical analysis

The *p*-values were determined using paired two-tailed Student’s *t*-tests using Microsoft Excel for Mac 2011 (Redmond, WA, USA). All the results were obtained using biologically independent replicates.

## Results

### The effect of salts on organic excretion; succinate and lactate production enhanced by K^+^

**Figure 1:**
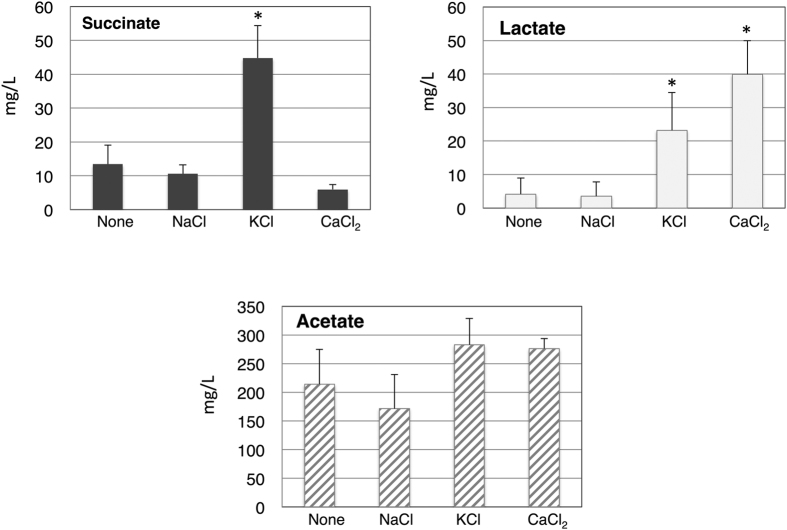
Levels of succinate, lactate, and acetate from the wild-type cyanobacterium *Synechocystis* 6803 under dark, anaerobic conditions. Organic acids excreted from cells incubated for 3 days under dark, anaerobic incubation were quantified by HPLC. Data represent means ± SD from four biological independent replicates. Each salt was added at 100 mM concentration. None designates in the absence of salt during dark, anaerobic incubation. Asterisks indicate statistically significant differences between samples in the absence and presence of KCl (Student’s *t*-test; *P < 0.05).

Organic acids are excreted from *Synechocystis* 6803 GT cells incubated in BG-11 medium or HEPES-buffer under dark, anaerobic conditions^10^. To test the effect of salts on organic acid production, cells were incubated in HEPES-buffer with/without various salts (each salt was added at 100 mM concentration) under dark, anaerobic conditions for 3 days. Succinate excretion was upregulated about 3 times in the presence of KCl (Fig. 1). Lactate levels increased 5.5 and 9.5 times by the addition of KCl and CaCl_2_, respectively (Fig. 1). Acetate levels were less affected by the addition of three different salts (Fig. 1). Acetate was most abundant among organic acids excreted under dark, anaerobic conditions for all experimental groups (Fig. 1). The ratios of succinate and lactate increased from 6% to 13% and from 2% to 7%, by KCl addition, respectively (Fig. 1). Lactate levels increased to 12% by CaCl_2_ addition (Fig. 1). Total organic acid production was 231.5 mg/L under salt free conditions, and 185.6, 351.0, and 322.1 mg/L in the presence of NaCl, KCl, and CaCl_2_, respectively (Fig. 1).

**Figure 2:**
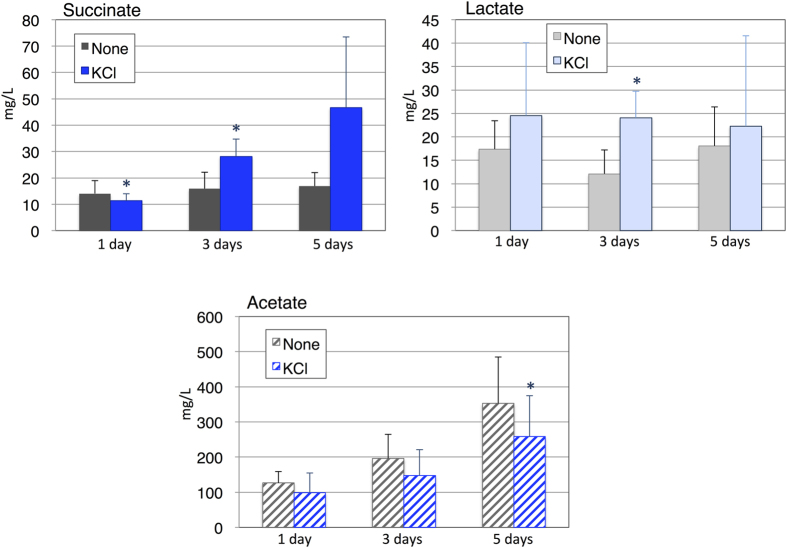
Time-course experiment measuring the organic acid excretion (succinate, lactate, and acetate). Organic acids excreted from cells incubated for 1, 3, or 5 days under dark, anaerobic incubation were quantified by HPLC. Data represent means ± SD from four biological independent replicates. None and KCl designates in the absence and presence of 100 mM KCl during dark, anaerobic incubation. Asterisks indicate statistically significant differences between samples in the absence and presence of KCl (Student’s *t*-test; *P < 0.05).

Time-course experiments revealed that succinate continued to increase under dark, anaerobic conditions for up to 5 days in the presence of KCl (Fig. 2). Lactate did not increase during 5 days of dark, anaerobic conditions regardless of K^+^ addition (Fig. 2). Acetate gradually increased after the prolonged incubation, and KCl addition reduced the acetate levels after 3 or 5 days in dark, anaerobic incubation (Fig. 2).

**Figure 3:**
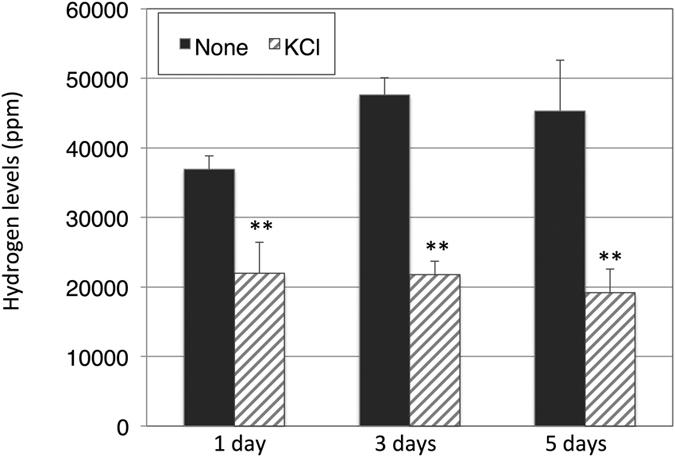
Levels of hydrogen accumulated under dark, anaerobic incubation for 1, 3, or 5 days. Hydrogen concentration was measured by GC-TCD. Data represent means ± SD from six to eight biological independent replicates. None and KCl designates in the absence and presence of 100 mM KCl under dark, anaerobic incubation, respectively. Asterisks indicate statistically significant differences between samples in the absence and presence of KCl (Student’s *t*-test; **P < 0.005).

In addition to organic acids, hydrogen also accumulates during dark, anaerobic conditions^24^, and the K^+^ effect on hydrogen production was examined. The hydrogen levels decreased in the presence of KCl under 1–5 days of dark, anaerobic conditions (Fig. 3).

### The additive effect by K^+^ and genetic manipulation on succinate and lactate production

**Figure 4:**
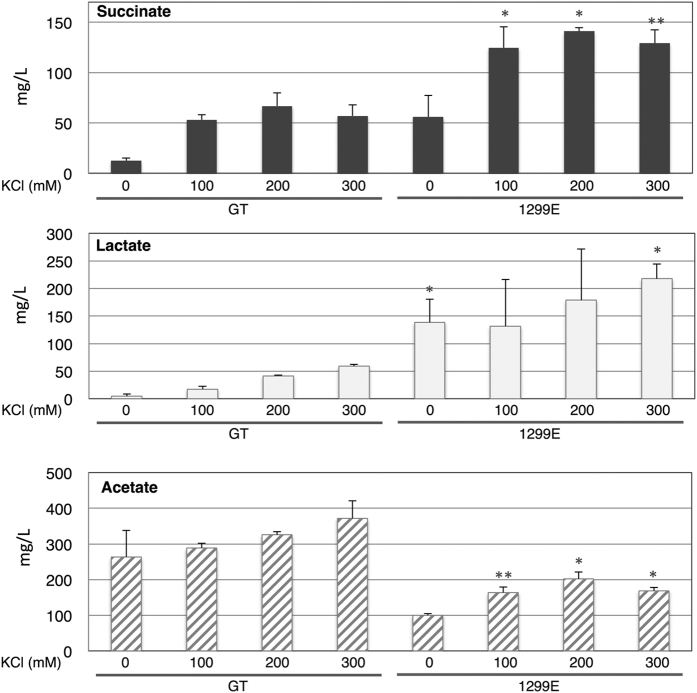
Production of organic acids from the cyanobacterium *Synechocystis* 6803 strain lacking *ackA* (sll1299) and overexpressing *sigE* under different KCl concentrations. Levels of organic acids excreted during 3 days of dark, anaerobic incubation were quantified by HPLC. The 1299E strain represents the strains lacking *ackA and* overexpressing *sigE*. Data represent means ± SD from three biological independent replicates. Asterisks indicate statistically significant differences between GT and 1299E (Student’s *t*-test; *P < 0.05, **P < 0.005).

Previous studies showed that the succinate production of the strain 1299E, an *ackA* knockout with overexpressed *sigE*, increased to 5 times that of the GT^10^; the effect of K^+^ was tested using this mutant. Both succinate and lactate production in the 1299E cells increased in the presence of KCl (Fig. 4). The maximum levels of succinate and lactate were 141.0 and 217.6 mg/L, respectively, which are 11 and 46 times that of the control (the levels of GT cells without salt) (Fig. 4). Acetate levels of the 1299E cells were lower than those of GT, both in the absence and presence of KCl (Fig. 4).

In contrast with the GT cells, lactate was the dominant organic acids excreted from 1299E cells (Fig. 4). The addition of 100 or 200 mM KCl increased the ratio of succinate and decreased the ratio of lactate in 1299E (Fig. 4). The total organic acid production reached more than 500 mg/L from 1299E cells in the presence of either 200 or 300 mM KCl, which was about 2 times that of the GT cells in the absence of KCl (Fig. 4). Additionally, we examined the effect of 100 mM CaCl_2_ on organic acid production in 1299E. CaCl_2_ reduced the succinate levels of 1299E to less than 20 mg/mL, and hardly affected the levels of lactate and acetate (Fig. S1).

### Metabolomic analysis revealing the increase in anionic metabolite biosynthesis by K^+^

To investigate the effect of K^+^ on primary metabolism, we measured glycogen and polyhydroxybutylate (PHB) levels before and after dark, anaerobic incubation. Decrease in glycogen under dark, anaerobic incubation was not affected by K^+^ (Table 1). PHB levels increased during dark, anaerobic incubation and the PHB biosynthesis was slightly enhanced in the presence of KCl, but the difference was not statistically significant (Table 1).

**Figure 5:**
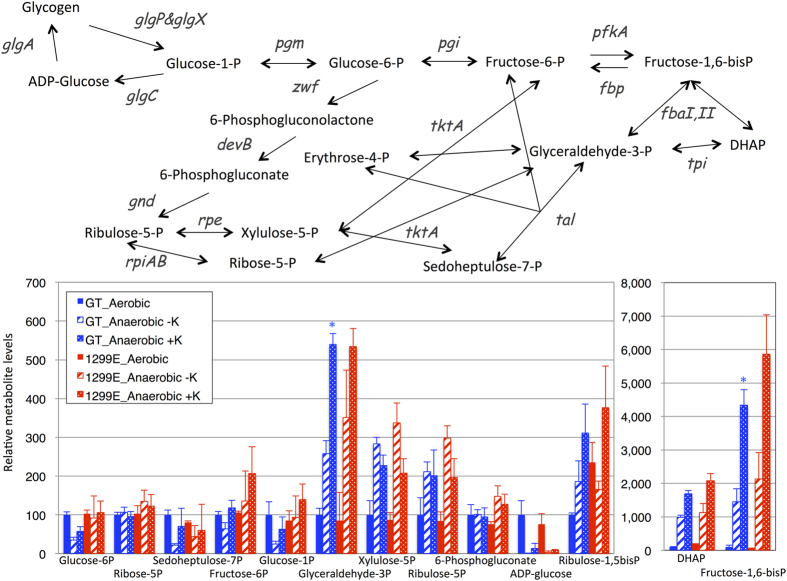
Levels of metabolites from the upper half of sugar metabolism of the cyanobacterium *Synechocystis* 6803 before and after dark, anaerobic incubation for 3 days. Data represent means ± SD from three biological independent replicates. Metabolite levels were calibrated relative to those of corresponding metabolites in the cells under aerobic conditions (set at 100%). Metabolite data without KCl were obtained from our previous data^10^. P designates phosphate. Asterisks indicate the statistically significant differences between samples in the absence and presence of KCl (Student’s *t*-test; *P < 0.05).

Metabolome analysis was performed using LC-MS/MS and GC-MS. Levels of metabolites from cells grown for 3 days under continuous light with the air containing 1% CO_2_ were defined as “metabolites under aerobic conditions”. Levels of metabolites from cells concentrated into 10 mL HEPES-buffer to be A_730_ = 20 in a vial, followed by purging air by nitrogen gas, and incubated for 3 days under dark, anaerobic conditions were defined as “metabolites under anaerobic conditions”. Sugar phosphates upstream of sugar metabolism such as glucose-6-phosphate, glucose-1-phosphate, ribose-5-phosphate, and fructose-6-phosphate, did not increase in the GT cells during dark, anaerobic incubation for 3 days, regardless of KCl addition (Fig. 5 and Table S1). Glyceraldehyde-3-phosphate and fructose-1,6-bisphosphate in the GT cells increased under dark anaerobic conditions, and the increases were upregulated in the presence of KCl (Fig. 5). The metabolite levels of upstream sugar metabolism were similar between the GT and 1299E strains (Fig. 5).

**Figure 6:**
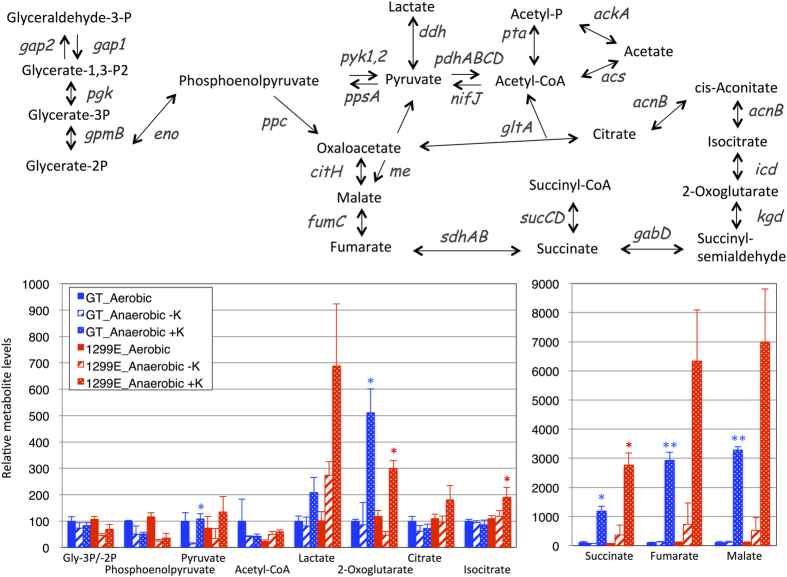
Levels of metabolites downstream of glycolysis, pyruvate metabolism, and the TCA cycle of the cyanobacterium *Synechocystis* 6803 before and after dark, anaerobic incubation for 3 days. Data represent means ± SD from three biological independent replicates. Metabolite levels were calibrated relative to those of corresponding metabolites in the cells under aerobic conditions (set at 100%). Metabolite data without KCl were obtained from our previous data^10^. P designates phosphate. Gly designates glycerate. Asterisks indicate the statistically significant differences between in the absence and presence of KCl (Student’s *t*-test; *P < 0.05).

The metabolite levels downstream of sugar metabolism were altered by KCl addition during dark, anaerobic incubation for 3 days (Fig. 6 and Table S1). Organic acids such as 2-oxoglutarate, succinate, fumarate, and malate increased by 5, 12, 30, and 33 times in the GT cells during dark, anaerobic incubation in the presence of KCl, respectively, compared to their levels under aerobic conditions (Fig. 6). The 1299E strain in particular accumulated higher levels of isocitrate and succinate than in the GT cells when incubated under dark, anaerobic conditions (Fig. 6).

## Discussion

In this study, we found that the primary metabolism of unicellular cyanobacterium could be altered by salt conditions; in particular, increased K^+^ upregulated the biosynthesis of anionic metabolites and the organic acid production of *Synechocystis* 6803 cells during dark, anaerobic incubation.

KCl increased succinate and lactate production, and CaCl_2_ increased lactate, but NaCl did not enhance organic acid production (Fig. 1). These results suggested that an increase in succinate and lactate was owing to the salt species, not the salt concentration and osmotic changes. Lactate is produced by lactate dehydrogenase, which is encoded by *ddh* (slr1556)^10^. Conversion between fumarate and succinate is usually a reversible reaction in both prokaryotic and eukaryotic cells. In the case of *E. coli*, succinate dehydrogenase oxidizes succinate to produce fumarate under aerobic conditions, and fumarate reductase reduces fumarate to produce succinate under anaerobic conditions^27^. Succinate dehydrogenase and fumarate reductase are homologous, and their enzymatic property could not be determined from their amino acid sequences^28^. Mammalian succinate dehydrogenase can catalyze both succinate oxidation and fumarate reduction^28^, and hence, the kinetic property of succinate dehydrogenase/fumarate reductase varies depending on the organism. Genetic analysis indicated that *Synechocystis* 6803 succinate dehydrogenase oxidizes plastoquinones, and a deficiency of succinate dehydrogenase decreases levels of both succinate and fumarate^29^. Therefore, succinate dehydrogenase is essential for succinate production, and determining its kinetic property is required to understand succinate and fumarate metabolism under dark, anaerobic conditions in this cyanobacterium. The catalytic activity of plant succinate dehydrogenase is negatively regulated by potassium^30^; therefore, K^+^ may be inclined to reduce fumarate rather than oxidize succinate. The effect of K^+^ on cyanobacterial succinate dehydrogenase should be further investigated.

Hydrogen production decreased with K^+^ under dark, anaerobic conditions (Fig. 3). Hydrogen is thought to function with electron valves to maintain redox status; it is closely related to primary metabolism in *Synechocystis* 6803^31^. Hydrogen, lactate and succinate production require NAD(P)H, and our metabolomic analysis revealed that NADPH disappeared under dark, anaerobic conditions regardless of K^+^ and strains (Table S1). Hydrogen and organic acid production may be competitive due to limited NADPH, and K^+^ increased the ratio of reductant consumption toward organic acid production. We have recently showed that the decrease in hydrogenase activity increased succinate and lactate production in *Synechocystis* 6803^32^, and K^+^ may decrease the hydrogenase activity. The negative correlation between hydrogen and organic acid biosynthesis is thus rational and may be important for succinate production using *Synechocystis* 6803. Negative correlation between succinate/lactate and acetate was remarkably observed in the 1299E mutant in the presence of K^+^ (Fig. 4), consistent with our previous results^10^.

Metabolome analysis revealed an increase in metabolite levels downstream of sugar metabolism (fructose-1,6-bisphosphate and glyceraldehyde-3-phosphate) in the presence of K^+^ under dark, anaerobic conditions (Fig. 5). Organic acids such as succinate, fumarate, malate, and 2-OG are also strikingly increased by adding K^+^ during dark, anaerobic incubation (Fig. 6). Here we first showed the potassium effect on *Synechocystis* 6803 primary metabolism: K^+^ massively enhanced the biosynthesis of anionic metabolites. One possible explanation for increased anionic metabolites is to balance ionic charges for increasing K^+^ concentration. In the case of *Vicia faba* L. stomata cells, malate and citrate are the counter ions of K^+^ during stomata opening^33^. Thus, organic acids and sugar phosphates may be counter anions of K^+^ in *Synechocystis* 6803. Another possibility is the changes in metabolic enzymes caused by K^+^. Phosphofructokinases of both prokaryotes and eukaryotes are activated in the presence of K^+^ 34,35. Generally, phosphofructokinase participates in a rate-limiting step of glycolysis, but pyruvate kinase activity is also important for determining the flux of sugar catabolism in eukaryotes^36^; pyruvate kinase is known to be activated by K^+ ^37. Thus, the coordinated regulation of these metabolic enzymes may determine the flux of sugar catabolism in this cyanobacterium.

The addition of K^+^ to the 1299E strain enhanced succinate and lactate production; the maximum amount in this study reached up to 141 and 218 mg/L, respectively (Fig. 4). The highest productivity of lactate in cyanobacteria is 1.84 g/L^38^, and therefore, further increase in lactate production is necessary through additional genetic engineering. In terms of succinate, this is the highest productivity in cyanobacteria without adding external carbon sources. By exploiting our results, succinate and lactate production are 84 and 132 kg/ha/year, respectively. The increase in succinate and lactate by *ackA* knockout/*sigE* overexpression and K^+^ addition were additive (Fig. 4), indicating that the K^+^ effect is different from the genetic manipulation of *ackA* and *sigE*. SigE is a positive regulator of glycogen and glucose catabolism, and *sigE* overexpression increases citrate and isocitrate, but decreases fumarate and malate under normal light and aerobic conditions^39^. Therefore, dark, anaerobic conditions with K^+^ increased the biosynthesis of the latter half of the TCA cycle metabolites regardless of *sigE* overexpression. Concerning heterotrophic bacteria, genetically engineered *Corynebacterium glutamicum* produced 146 g/L succinate using glucose and sodium bicarbonate as carbon sources^40^. 50 g dry cell weight/L of *C. glutamicum* cells were used for the maximum succinate production level^39^, and in our experiment, the dry cell weight was approximately 4.5 g/L (calculated using 20 mL cell cultures with A_730_ = 20) to produce the maximum levels (Fig. 4). Thus, the succinate production rate per dry cell weight by direct conversion of CO_2_ using cyanobacteria is about 100 times less than that of *C. glutamicum* using glucose as a carbon source. Further metabolic engineering of direct CO_2_ conversion may increase organic acid production, which requires a better understanding of the primary metabolism of cyanobacteria.

## Additional Information

**How to cite this article**: Ueda, S. *et al*. Anionic metabolite biosynthesis enhanced by potassium under dark, anaerobic conditions in cyanobacteria. *Sci. Rep.* **6**, 32354; doi: 10.1038/srep32354 (2016).

## Supplementary Material

Supplementary Information

Supplementary Information

## Figures and Tables

**Table 1:** Relative glycogen and PHB levels before and after dark, anaerobic incubation.

Strain	Aerobic	Anaerobic without KCl	Anaerobic with KCl
Glycogen	100 ± 11.6	81.8 ± 9.1	79.1 ± 3.6
PHB	100 ± 52.1	185.8 ± 53.5	243.3 ± 51.4

Data represent means ± SD results from six independent experiments for glycogen and three independent experiments for PHB, respectively. Glycogen and PHB levels were calibrated relative to that in under light, aerobic conditions (set at 100%). Anaerobic indicates glycogen and PHB levels of the cells incubated for three days under dark, anaerobic conditions.

## References

[b1] Bullerjahn G. S. & Post A. F. Physiology and molecular biology of aquatic cyanobacteria. Front. Microbiol. 5, 359 (2014). 25076944 10.3389/fmicb.2014.00359 PMC4099938

[b2] Berla B. M. . Synthetic biology of cyanobacteria: unique challenges and opportunities. Front. Microbiol. 4, 246 (2013). 24009604 10.3389/fmicb.2013.00246 PMC3755261

[b3] Branco dos Santos F., Du W. & Hellingwerf K. J. *Synechocystis*: not just a plug-bug for CO_2_, but a green *E. coli*. Front. Bioeng. Biotechnol. 2, 36 (2014). 25279375 10.3389/fbioe.2014.00036 PMC4166995

[b4] Stal L. J. & Moezelaar R. Fermentation in cyanobacteria. FEMS Microbiol. Rev. 21, 179–211 (1997).

[b5] Kothari A., Parameswaran P. & Garcia-Pichel F. Powerful fermentative hydrogen evolution of photosynthate in the cyanobacterium *Lyngbya aestuarii* BL J mediated by a bidirectional hydrogenase. Front. Microbiol. 5, 680 (2014). 25540642 10.3389/fmicb.2014.00680 PMC4261827

[b6] Tamagnini P. . Cyanobacterial hydrogenases: diversity, regulation and application. FEMS Microbiol. Rev. 31, 692–720 (2007). 17903205 10.1111/j.1574-6976.2007.00085.x

[b7] Appel J., Phunpruch S., Steinmüller K. & Schulz R. The bidirectional hydrogenase of *Synechocystis* sp. PCC 6803 works as an electron valve during photosynthesis. Arch. Microbiol. 173, 333–338 (2000). 10896211 10.1007/s002030000139

[b8] McNeely K., Xu Y., Bennette N., Bryand D. A. & Dismukes G. C. Redirecting reductant flux into hydrogen production via metabolic engineering of fermentative carbon metabolism in a cyanobacterium. Appl. Environ. Microbiol. 76, 5032–5038 (2010). 20543051 10.1128/AEM.00862-10 PMC2916493

[b9] Carrieri D., Ananyev G., Lenz O., Bryant D. A. & Dismukes G. C. Contribution of a sodium ion gradient to energy conservation during fermentation in the cyanobacterium *Arthrospira* (*Spirulina*) *maxima* CS-328. Appl. Environ. Microbiol. 77, 7185–7194 (2011). 21890670 10.1128/AEM.00612-11 PMC3194876

[b10] Osanai T. . Genetic manipulation of a metabolic enzyme and a transcriptional regulator increasing succinate excretion from unicellular cyanobacterium. Front. Microbiol. 6, 1064 (2015). 26500619 10.3389/fmicb.2015.01064 PMC4594341

[b11] Angermayr S. A., Paszota M. & Hellingwerf K. J. Engineering a cyanobacterial cell factory for production of lactic acid. Appl. Environ. Microbiol. 78, 6908–6913 (2012). 22865063 10.1128/AEM.01587-12 PMC3457509

[b12] Angermayr S. A. . Exploring metabolic engineering design principles for the photosynthetic production of lactic acid by *Synechocystis* sp. PCC6803. Biotechnol. Biofuels. 7, 99 (2014). 24991233 10.1186/1754-6834-7-99 PMC4078008

[b13] Tay Y.-F., Ho C.-H., Chen H.-Y. & Lin S.-H. Integration of nitrogen and potassium signaling. Annu. Rev. Plant Biol. 62, 207–226 (2011). 21495843 10.1146/annurev-arplant-042110-103837

[b14] Richey B. . Variability of the intracellular ionic environment of *Escherichia coli*. Differences between *in vitro* and *in vivo* effects of ion concentrations on protein-DNA interactions and gene expression. J. Biol. Chem. 262, 7157–7164 (1987). 3108249

[b15] Epstein W. The roles and regulation of potassium in bacteria. Prog. Nucl. Acid Res. Mol. Biol. 75, 293–320 (2003). 10.1016/s0079-6603(03)75008-9 14604015

[b16] Chérel I., Lefoulon C., Boeglin M. & Sentenac H. Molecular mechanisms involved in plant adaptation to low K^+^ availability. J. Exp. Bot. 65, 833–848 (2014). 24293613 10.1093/jxb/ert402

[b17] Page M. J. & Cera E. D. Role of Na^+^ and K^+^ in enzyme function. Physiol. Rev. 86, 1049–1092 (2006). 17015484 10.1152/physrev.00008.2006

[b18] Checchetto V. . Thylakoid potassium channel is required for efficient photosynthesis in cyanobacteria. Proc. Natl. Acad. Sci. USA 109, 11043–11048 (2012). 22711813 10.1073/pnas.1205960109 PMC3390830

[b19] Alahari A. & Apte S. K. Pleiotropic effects of potassium deficiency in a heterocystous, nitrogen-fixing cyanobacterium, Anabaena torulosa. Microbiology 144, 1557–1563 (1998). 9639926 10.1099/00221287-144-6-1557

[b20] Nanatani K. . Comparative analysis of *kdp* and *ktr* mutants reveals distinct roles of the potassium transporters in the model cyanobacterium *Synechocystis* sp. strain PCC 6803. J. Bacteriol. 197, 676–687 (2015). 25313394 10.1128/JB.02276-14 PMC4334184

[b21] Williams J. G. K. Construction of specific mutations in photosystem II photosynthetic reaction center by genetic engineering methods in *Synechocystis* 6803. Methods Enzymol. 167, 766–778 (1988).

[b22] Rippka R. Isolation and purification of cyanobacteria. Methods Enzymol. 167, 3–27 (1988). 3148836 10.1016/0076-6879(88)67004-2

[b23] Kanesaki Y. . Identification of substrain-specific mutations by massively parallel whole-genome resequencing of *Synechocystis* sp. PCC 6803. DNA Res. 19, 67–79 (2012). 22193367 10.1093/dnares/dsr042 PMC3276265

[b24] Osanai T. . Pleiotropic effect of *sigE* over-expression on cell morphology, photosynthesis and hydrogen production in *Synechocystis* sp. PCC 6803. Plant J. 76, 456–465 (2013). 23941239 10.1111/tpj.12310

[b25] Osanai T. . Capillary electrophoresis-mass spectrometry reveals the distribution of carbon metabolites during nitrogen starvation in *Synechocystis* sp. PCC 6803. Environ. Microbiol. 16, 512–524 (2014). 23796428 10.1111/1462-2920.12170

[b26] Osanai T. . Alteration of cyanobacterial sugar and amino acid metabolism by overexpression *hik8*, encoding a KaiC-associated histidine kinase. Environ. Microbiol. 17, 2430–2440 (2015). 25403325 10.1111/1462-2920.12715

[b27] Hirsch C. A., Rasminsky M., Davis B. D. & Lin E. C. A fumarate reductase in *Escherichia coli* distinct from succinate dehydrogenase. J. Biol. Chem. 238, 3770–3774 (1963). 14109218

[b28] Cecchini G., Schröder I., Gunsalus R. P. & Maklashina E. Succinate dehydrogenase and fumarate reductase from *Escherichia coli*. Biochim. Biophy. Acta. 155, 3140–157 (2002). 10.1016/s0005-2728(01)00238-9 11803023

[b29] Cooley J. W. & Vermaas W. F. J. Succinate dehydrogenase and other respiratory pathways in thylakoid membranes of *Synechocystis* sp. strain PCC 6803: Capacity comparisons and physiological function. J. Bacteriol. 183, 4251–4258 (2001). 11418566 10.1128/JB.183.14.4251-4258.2001 PMC95315

[b30] Affourtit C., Krab K., Leach G. R., Whitehouse D. G. & Moore A. L. New insights into the regulation of plant succinate dehydrogenase. J. Biol. Chem. 276, 32567–32574 (2001). 11350973 10.1074/jbc.M103111200

[b31] Pinto F. . Construction of a chassis for hydrogen production. Physiological and molecular characterization of a *Synechocystis* sp. PCC 6803 mutant lacking a functional bidirectional hydrogenase. Microbiology 158, 448–464 (2012). 22096147 10.1099/mic.0.052282-0

[b32] Iijima H. . Metabolomics-based analysis revealing the alteration of primary carbon metabolism by the genetic manipulation of a hydrogenase HoxH in *Synechocystis* sp. PCC 6803. Algal Res. 18, 305–313 (2016).

[b33] Outlaw W. H. & Lowry O. H. Organic acid and potassium accumulation in guard cells during stomatal opening. Proc. Natl. Acad. Sci. USA 74, 4434–4438 (1977). 16592449 10.1073/pnas.74.10.4434 PMC431957

[b34] Mavis R. D. & Stellwagen E. The role of cations in yeast phosphofructokinase catalysis. J. Biol. Chem. 245, 674–680 (1970). 4244536

[b35] Stellwagen E. & Thompson S. T. Activation of *Thermus* phosphofructokinase by monovalent cations. Biochim. Biophys. Acta. 569, 6–12 (1979). 157165 10.1016/0005-2744(79)90075-5

[b36] Boscá L. & Corredor C. Is phosphofructokinase the rate-limiting step of glycolysis? Trends Biochem. Sci. 9, 372–373 (1984).

[b37] Oria-Hernández J., Cabrera N., Pérez-Montfort R. & Ramírez-Silva L. Pyruvate kinase revisited: the activating effect of K^+^. J. Biol. Chem. 280, 37924–37929 (2005). 16147999 10.1074/jbc.M508490200

[b38] Angermayr S. A. & Hellingwerf K. J. On the use of metabolic control analysis in the optimization of cyanobacterial biosolar cell factories. J. Phys. Chem. B 117, 11169–11175 (2013). 23506247 10.1021/jp4013152

[b39] Osanai T. . Genetic engineering of group 2 sigma factor SigE widely activates expressions of sugar catabolic genes in *Synechocystis* species PCC 6803. J. Biol. Chem. 286, 30962–30971 (2011). 21757761 10.1074/jbc.M111.231183 PMC3162455

[b40] Okino S. . An efficient succinic acid production process in a metabolically engineered *Corynebacterium glutamicum* strain. Appl. Microbiol. Biotechnol. 81, 459–464 (2008). 18777022 10.1007/s00253-008-1668-y

