# Supplementary material for: Anionic metabolite biosynthesis enhanced by potassium under dark, anaerobic conditions in cyanobacteria
Source: Sci Rep. 2016 Aug 31;6:32354. doi: 10.1038/srep32354 (PMC5006033; doi:10.1038/srep32354)

**Anionic metabolite biosynthesis enhanced by potassium under dark, anaerobic conditions in cyanobacteria**

**Sakiko Ueda^1^*, Yuki Kawamura^1^*, Hiroko Iijima^1^, Mitsuharu Nakajima^1^, Tomokazu Shirai^2^, Mami Okamoto^2^, Akihiko Kondo^2,3^, Masami Yokota Hirai^2^, Takashi Osanai^1,2^**

^1^School of Agriculture, Meiji University, 1-1-1, Higashimita, Tama-ku, Kawasaki, Kanagawa 214-8571, Japan

^2^RIKEN Center for Sustainable Resource Science, 1-7-22 Suehiro-cho, Tsurumi-ku, Yokohama, Kanagawa 230-0045, Japan

**^3^**Department of Chemical Science and Engineering, Graduate School of Engineering, Kobe University, 1-1, Rokkodai, Nada, Kobe 657-8501, Japan

Address correspondence to: Takashi Osanai, 1-1-1 Higashimita, Tama-ku, Kawasaki, Kanagawa 214-8571, Japan

*These authors contributed equally to this work.

Tel: +81-44-934-7103. Fax: +81-44-934-7103. E-mail: [tosanai@meiji.ac.jp](mailto:tosanai@meiji.ac.jp)

Running title: Anionic metabolite biosynthesis enhanced by K^+^

**Figure S1.** Production of organic acids from the cyanobacterium *Synechocystis* 6803 strain lacking *ackA* (sll1299) and overexpressing *sigE* in the presence of 100 mM CaCl_2_. Levels of organic acids excreted during 3 days of dark, anaerobic incubation were quantified by HPLC. The 1299E strain represents the strains lacking *ackA and* overexpressing *sigE*. Data represent means ± SD from biological independent replicates (*n* = 3~6). Asterisks indicate statistically significant differences between in the absence and presence of CaCl_2_ (Student’s *t*-test; **P* < 0.05, ***P* < 0.005).

Fig. S1


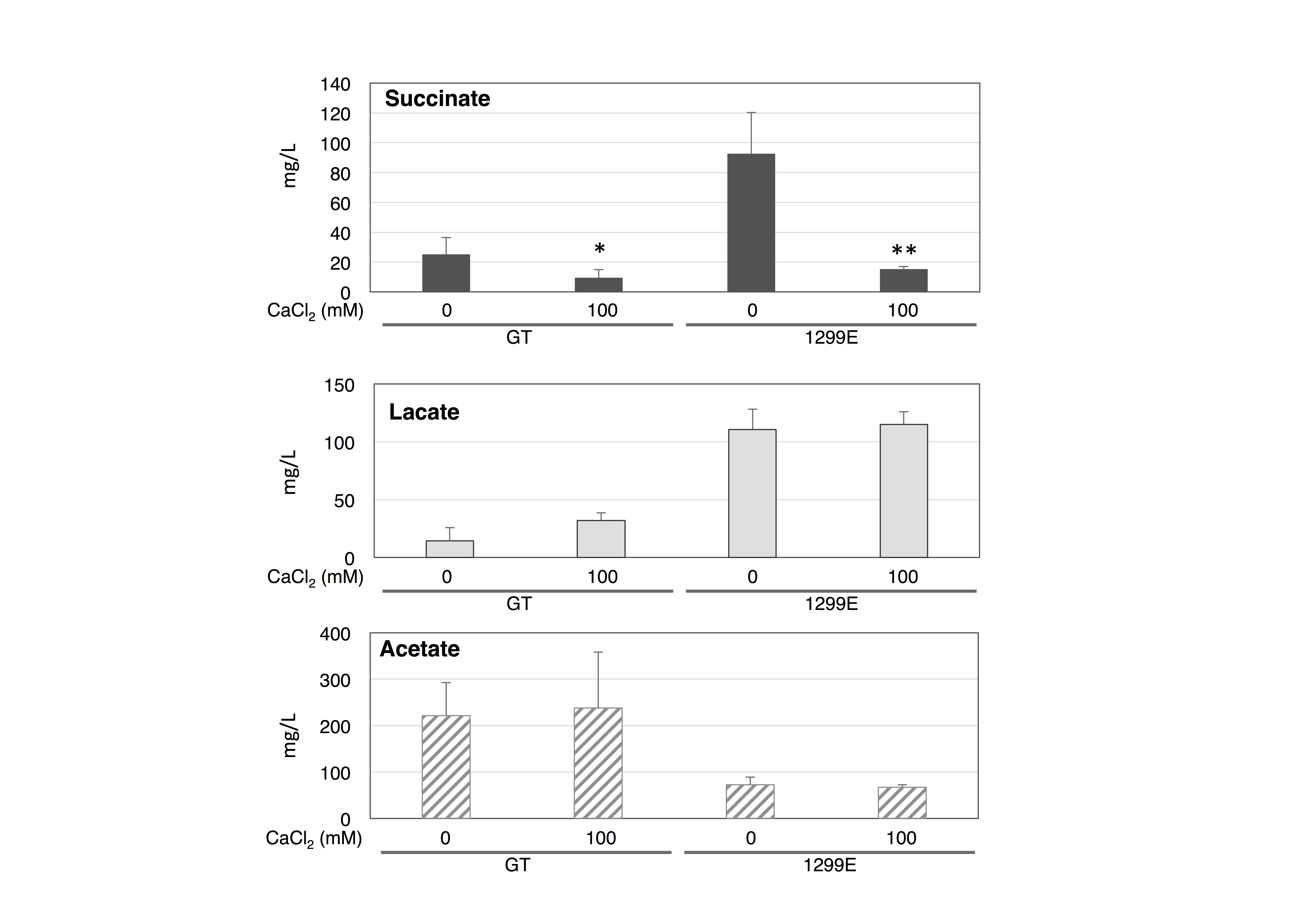

Supplement: Supplementary Information [file srep32354-s1.doc]
